# Excessive Cellular *S*-nitrosothiol Impairs Endocytosis of Auxin Efflux Transporter PIN2

**DOI:** 10.3389/fpls.2017.01988

**Published:** 2017-11-23

**Authors:** Min Ni, Lei Zhang, Ya-Fei Shi, Chao Wang, Yiran Lu, Jianwei Pan, Jian-Zhong Liu

**Affiliations:** ^1^College of Chemistry and Life Sciences, Zhejiang Normal University, Jinhua, China; ^2^Ministry of Education Key Laboratory of Cell Activities and Stress Adaptations, School of Life Sciences, Lanzhou University, Lanzhou, China

**Keywords:** endocytosis, nitric oxide, PIN-FORMED (PIN) proteins, polar auxin transport, *S*-nitrosoglutathione reductase

## Abstract

*S*-nitrosoglutathione reductase (GSNOR1) is the key enzyme that regulates cellular levels of *S*-nitrosylation across kingdoms. We have previously reported that loss of GSNOR1 resulted in impaired auxin signaling and compromised auxin transport in Arabidopsis, leading to the auxin-related morphological phenotypes. However, the molecular mechanism underpinning the compromised auxin transport in *gsnor1-3* mutant is still unknown. Endocytosis of plasma-membrane (PM)-localized efflux PIN proteins play critical roles in auxin transport. Therefore, we investigate whether loss of GSNOR1 function has any effects on the endocytosis of PIN-FORMED (PIN) proteins. It was found that the endocytosis of either the endogenous PIN2 or the transgenically expressed PIN2-GFP was compromised in the root cells of *gsnor1-3* seedlings relative to Col-0. The internalization of PM-associated PIN2 or PIN2-GFP into Brefeldin A (BFA) bodies was significantly reduced in *gsnor1-3* upon BFA treatment in a manner independent of *de novo* protein synthesis. In addition, the exogenously applied GSNO not only compromised the endocytosis of PIN2-GFP but also inhibited the root elongation in a concentration-dependent manner. Taken together, our results indicate that, besides the reduced PIN2 level, one or more compromised components in the endocytosis pathway could account for the reduced endocytosis of PIN2 in *gsnor1-3*.

## Introduction

Nitric oxide (NO) is a reactive free radical gaseous molecule that is involved in battery of biological processes both in animals and plants (Wendehenne et al., 2014). In plants, NO participates in biological processes such as stomatal closure, cell death and disease resistance, abiotic stress, flowering, and many other processes (Durner et al., 1998; Klessig et al., 2000; Neill et al., 2002; Lamattina et al., 2003; He et al., 2004, 2012; Wendehenne et al., 2004, 2014; Zeidler et al., 2004; Lee et al., 2008; Xuan et al., 2010; Fan and Liu, 2012; Lin et al., 2012; Ye et al., 2012; Mur et al., 2013).

*S*-nitrosylation, adding NO moiety to a protein, is a novel mechanisms by which NO regulates protein functions (Hess and Stamler, 2012; Wendehenne et al., 2014). This non-enzymatic reversible protein modification is analogous to protein phosphorylation (Stamler et al., 1992; Hess et al., 2005). Many proteins have been identified as targets of *S*-nitrosylation and their functions are regulated by this modification (Lindermayr et al., 2005; Forrester et al., 2009; Hess and Stamler, 2012; Yang et al., 2015). In plants, the target cysteine residues of some *S*-nitrosylated proteins have been identified and the functional importance of this modification is unraveled (Lindermayr et al., 2006, 2010; Belenghi et al., 2007; Romero-Puertas et al., 2007; Serpa et al., 2007; Tada et al., 2008; Chen et al., 2009; Wang et al., 2009, 2015; Yun et al., 2011; Astier et al., 2012; Feng et al., 2013; Yang et al., 2015; Hu et al., 2017; Liu et al., 2017).

The level of cellular protein *S*-nitrosylation is dynamic and governed by NO levels and de-nitrosylation catalyzed by *S*-nitrosoglutathione reductase (GSNOR) (Liu et al., 2001; Feechan et al., 2005) and thioredoxin (Tada et al., 2008; Benhar et al., 2009; Sengupta and Holmgren, 2012). GSNOR is the key enzyme controlling *S*-nitrosoglutathione (GSNO) levels by reducing GSNO to oxidized GSH and NH_3_ and thus indirectly controls the cellular levels of *S*-nitrosylated proteins (Liu et al., 2001, 2004; Feechan et al., 2005).

Auxin is one of mostly studied plant hormone that plays diverse roles in development (Teale et al., 2006). Auxin gradients, which are created and maintained by groups of transporters localized on plasma membrane (PM) are critical to auxin functions in the regulation of stem cell differentiation, the initiation of lateral organs and gravitropic responses (Woodward and Bartel, 2005; Leyser, 2006; Petrásek and Friml, 2009). One of the most important transporters is the PIN-FORMED (PIN) family of auxin efflux proteins (Chen et al., 1998; Galweiler et al., 1998; Muller et al., 1998; Geldner et al., 2001; Blilou et al., 2005; Wisniewska et al., 2006; Pan et al., 2009).

Clathrin-mediated endocytosis (CME) is an evolutionally conserved pathway that plays a critical role in determining protein abundance at the PM and/or the trans-Golgi network (TGN) during signaling transductions and retargeting/degradation of proteins at PM (Chen et al., 2011; McMahon and Boucrot, 2011; Wang et al., 2013). CME is the predominant pathway for the internalization of numerous membrane-localized proteins including PINs (Paciorek et al., 2005). By inhibiting the endocytosis of PIN, auxin increases levels of various PINs at the PM (Paciorek et al., 2005). As a result, auxin promotes its own efflux by vesicle-trafficking-dependent mechanism (Paciorek et al., 2005). In addition to CME, a BFA-insensitive and clathrin-independent endocytic route has also been reported for PM resident proteins (Beck et al., 2012).

We have previously reported that loss of GSNOR1 function in Arabidopsis impairs both auxin signaling and polar auxin transport and thereby the *gsnor1-3* mutant displays multiple auxin-related morphological defects including short and highly branched statures, short primary roots, and lack of lateral roots. The compromised polar auxin transport in *gsnor1-3* is due to universally reduced levels of auxin efflux transporters PIN proteins at the plasma membrane (PM) (Shi et al., 2015). However, whether loss of GSNOR1 inhibits polar auxin transport exclusively through reducing the abundance of PINs at PM or additional mechanisms are also involved, are largely unknown. Here, we showed that loss of GSNOR1 inhibited the internalization of either the transgenically expressed PIN2-GFP or the endogenous PIN2 independent of *de novo* protein synthesis and this inhibition could be recapitulated by exogenously applied GSNO. Furthermore, similar to loss of GSNOR1, exogenously applied GSNO inhibited the root elongation in a concentration dependent manner. Together, our results reveal an additional layer of complex roles of NO in regulating plant growth and development through modulating internalization of auxin efflux transporter.

## Results

### Loss of GSNOR1 results in reduced internalization of transgenically expressed PIN2-GFP

**Figure 1:**
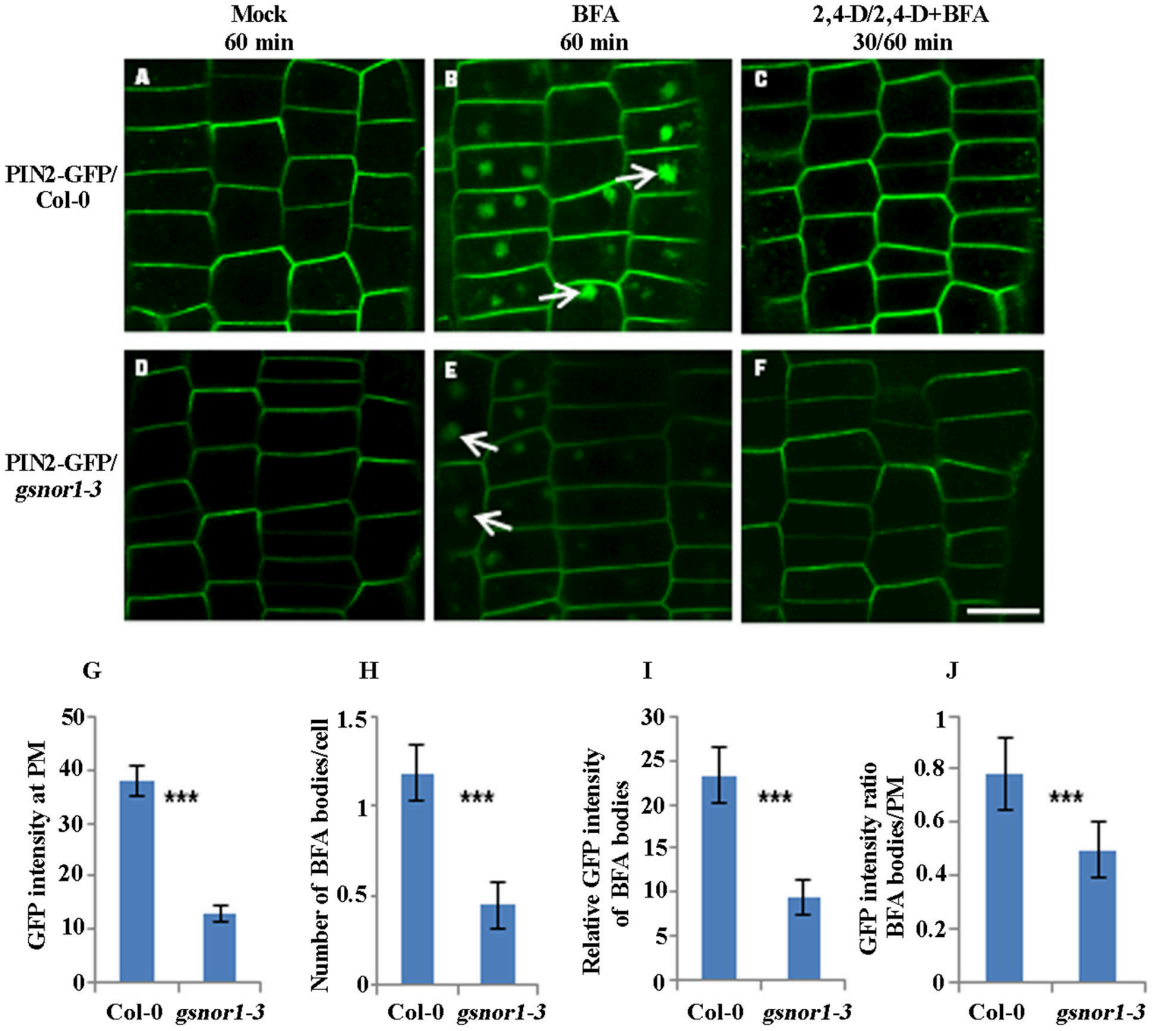
The internalization and not the auxin inhibition of internalization of the transgenically expressed PIN2-GFP is impaired in *gsnor1-3* mutant. Six-day-old *ProPIN2*:*PIN2-GFP*-expressing transgenic seedlings either in Col-0 or in *gsnor1-3* background were treated with Mock **(A,D)**, 50 μM BFA for 60 min **(B,E)**, and with 10 μM 2, 4-D for 30 min, followed by treatment with 50 μM BFA+ 10 μM 2, 4-D for additional 60 min **(C,F)**. Images were captured by confocal laser scanning microscopy (CLSM, Leica TCS SP5 AOBS). The numbers of BFA bodies (see white arrows) were counted and the fluorescence intensities both at the BFA bodies and at the PM were measured, respectively, using Image J (http://rsb.info.nih.gov/ij) and the statistical data were summarized **(G–J)**. **(G)** GFP intensity at the PM; **(H)** Number of BFA bodies per cell; **(I)** Relative GFP intensities of the BFA bodies; **(J)** The GFP intensity ratios of the BFA bodies/PM. Bar = 50 μm. ^***^Indicates significant differences between Col-0 and *gsnor1-3* by Student's *t*-test at 0.001 level.

To examine the effect of NO signaling on the internalization of the PM-associated PIN proteins, we used Brefeldin A (BFA; 50 μM), a vesicle trafficking inhibitor (Geldner et al., 2001), to visualize the PIN2-GFP internalization in the wild-type and *gsnor1-3* seedlings that express the *PIN2-GFP* driven by its native promoter (*ProPIN2*:*PIN2-GFP*). Consistent with our previous report (Shi et al., 2015), the intensity of the PIN2-GFP fluorescence at the PM was significantly reduced in *gsnor1-3* mutants relative to the wild-type cells under mock conditions (compare Figures 1A,D; and Figure 1G). As expected, both the numbers and the relative intensities of the PIN2-GFP-labeled BFA bodies were also significantly reduced in the *gsnor1-3* compared to the wild-type cells (compare white arrow-pointed BFA bodies in Figures 1B,E; and see statistical data in Figures 1H,I), indicating that the internalization of the PIN2-GFP was reduced in th*e gsnor1-3* mutants. To dissect whether the reduced numbers and intensities of the BFA-induced PIN2-GFP fluorescent bodies in *gsnor1-3* is exclusively resulted from the reduced levels of PIN2-GFP at the PM, we further analyzed the ratios of GFP signals in BFA bodies to those at the PM both in Col-0 and *gsnor1-3*, respectively, after BFA treatment. As shown in Figure 1J, the GFP signal ratio of BFA bodies/PM was significantly lower in *gsnor1-3* mutants than in the wild-type cells, indicating that, besides the reduced level of the PIN2-GFP at the PM, PIN2-GFP internalization itself is also compromised in the *gsnor1-3* mutant seedlings.

**Figure 2:**
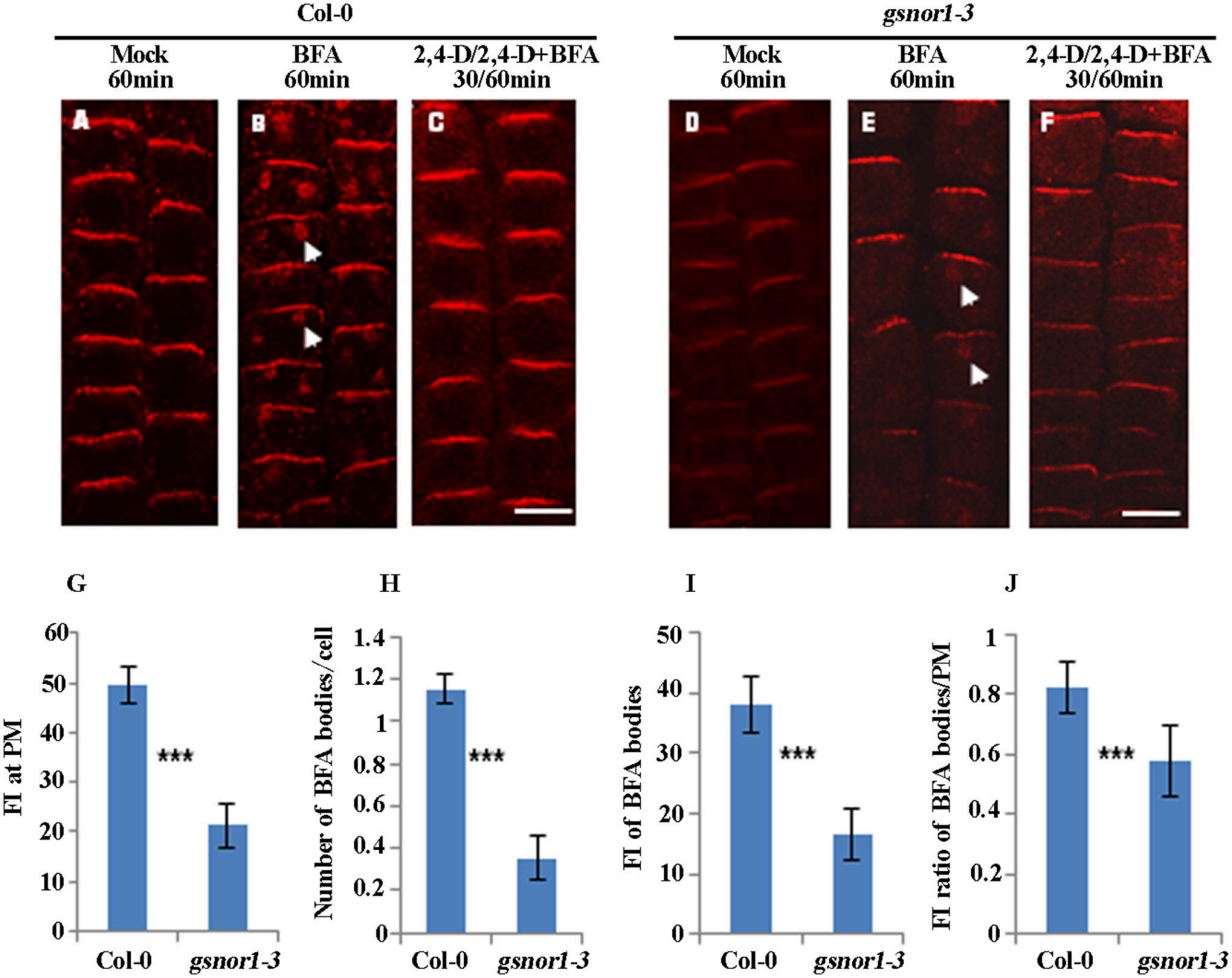
The internalization and not the auxin inhibition of internalization of the endogenous PIN2 is impaired in *gsnor1-3* mutant. The immunolocalizations were performed using PIN2-specific antibodies (Wang et al., 2013) and Cy3-labeled anti-rabbit secondary antibodies (Sigma-Aldrich) on the primary roots of 6-day-old seedlings treated with Mock **(A,D)**, 50 μM BFA for 60 min **(B,E)** or with 10 μM 2, 4-D for 30 min, followed by treatment with 50 μM BFA+ 10 μM 2, 4-D for additional 60 min **(C,F)**. Images were captured using CLSM (Leica TCS SP5 AOBS). The numbers of BFA bodies (see white arrows) were counted and the fluorescence intensities (FI) at the BFA bodies and at the PM were measured, respectively, using Image J (http://rsb.info.nih.gov/ij) and the statistical data were summarized **(G–J)**. **(G)** Fluorescence intensity (FI) at the PM; **(H)** Number of BFA bodies per cell; **(I)** Relative fluorescence intensities (FI) of the BFA bodies; **(J)** The fluorescence intensity (FI) ratios of the BFA bodies/PM. Bar = 50 μm. ^***^Indicates significant differences between Col-0 and *gsnor1-3* by Student's *t*-test at 0.001 level.

Auxin inhibits internalization of PM proteins (Paciorek et al., 2005). To address whether auxin inhibitory effect on PIN2 endocytosis is altered in the *gsnor1-3* mutants, we treated the transgenic seedlings expressing the PIN2-GFP both in the Col-0 and the *gsnor1-3* firstly with 10 μM 2,4-D for 30 min and followed by treatment with 10 μM 2,4-D plus 50 μM BFA for additional 60 min as described (Wang et al., 2013). As shown in Figure 1, 2,4-D similarly blocked the PIN2-GFP internalization both in the wild-type and the mutant cells (compare 1C and 1F), indicating that auxin inhibition of PIN2 internalization is not significantly impaired in the *gsnor1-3* mutant.

### Loss of GSNOR1 results in reduced internalization of the endogenous PIN2

Next, to test whether the internalization of endogenous PIN2 is similarly impaired as PIN2-GFP in the *gsnor1-3* mutant, we performed immunofluorescence microscopy analysis using affinity-purified anti-PIN2-specific antibodies (Wang et al., 2013). Similar to the PIN2-GFP shown in Figure 1, the level of the PM-localized endogenous PIN2 was significantly reduced in the *gsnor1-3* cells compared to the wild-type cells under the mock conditions (compare Figures 2A,D and Figure 2G). Similarly, the numbers and the relative intensities of PIN2-labeled BFA bodies (compare the white arrow-pointed BFA bodies in Figures 2B,E; also see statistical data in Figures 2H,I) and the fluorescence intensity ratio of BFA bodies/PM of the endogenous PIN2 (Figure 2J) were all significantly decreased in the *gsnor1-3* mutant compared to the wild-type cells. Again, the inhibition of the PIN2 internalization in the presence of 2,4-D was not significantly altered in the *gsnor1-3* mutant compared to the Col-0 cells (Figures 2C,F, no visible BFA bodies). These results confirmed the conclusions drawn from the studies using the *ProPIN2*:*PIN2-GFP*-expressing transgenic seedlings (Figure 1), suggesting that the transgenic expressed PIN2-GFP driven by its own promoter can recapitulate the endogenous PIN2.

### Loss of GSNOR1 results in the reduced endocytosis of PIN2-GFP in the absence of *de novo* protein synthesis

**Figure 3:**
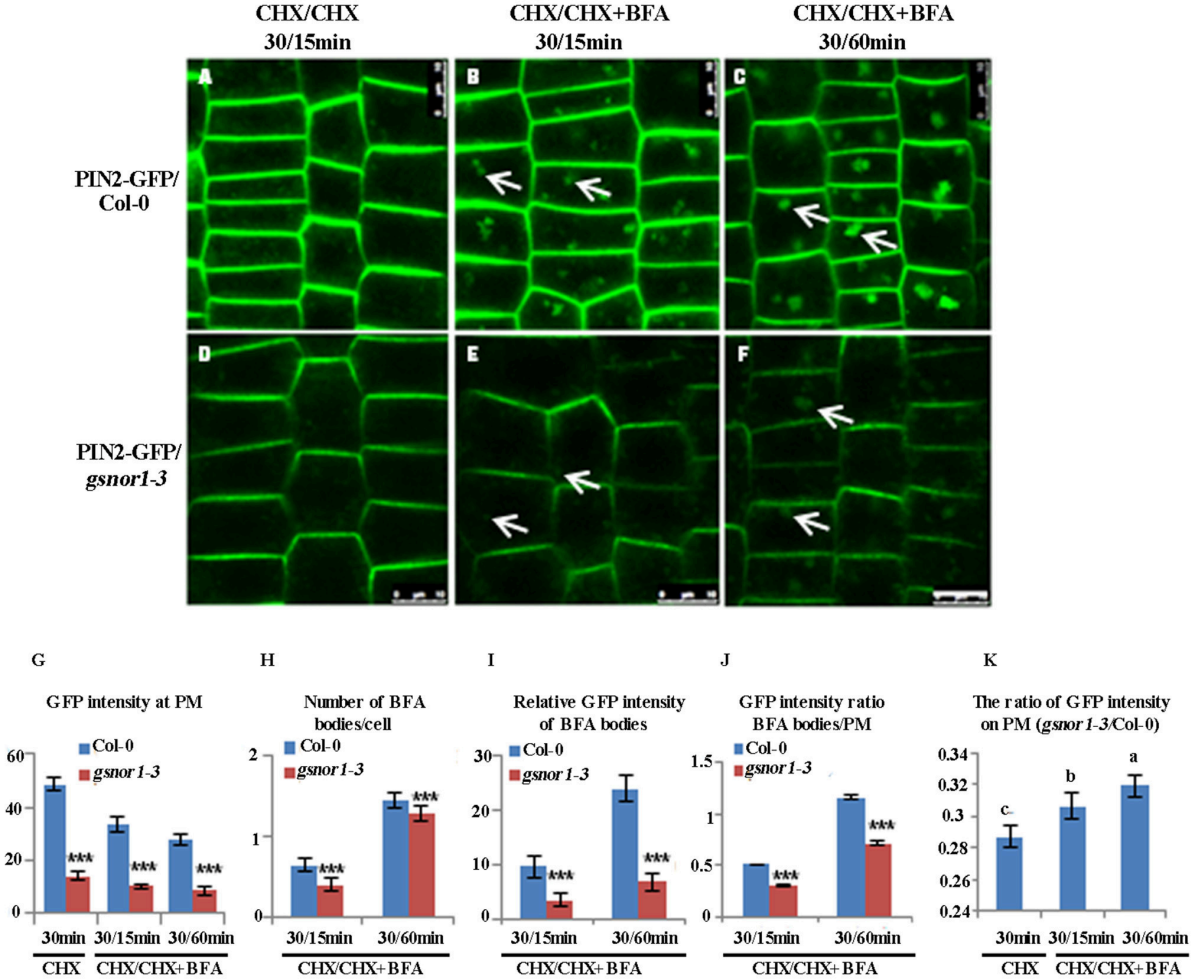
The internalization of PIN2-GFP is reduced in *gsnor1-3* in the absence of *de novo* protein synthesis. *ProPIN2*:*PIN2-GFP* transgenic Col-0 and *gsnor1-3* mutant seedlings (6-day-old) were treated with cycloheximide (CHX) for 30 min first, followed by CHX alone for 15 min **(A,D)** or co-treatment with CHX+BFA for 15 **(B,E)** or 60 min **(C,F)**. Images were captured by confocal laser scanning microscopy (CLSM, Leica TCS SP5 AOBS). The numbers of BFA bodies (see white arrows) were counted and the fluorescence intensities at the BFA bodies as well as at the PM were measured, respectively, using Image J (http://rsb.info.nih.gov/ij) and the statistical data were summarized **(G**–**K)**. **(G)**. GFP intensity at the PM; **(H)** Number of BFA bodies per cell; **(I)** Relative GFP intensities of the BFA bodies; **(J)** GFP intensity ratios of the BFA bodies/PM; **(K)** GFP intensity ratios on PM (*gsnor1-3*/Col-0). Bar = 50 μm. ^***^Indicates significant differences between Col-0 and *gsnor1-3* by Student's *t*-test at 0.001 level. a, b, and c Indicate significant differences between the treatments by Student's *t*-test.

To accurately assess the effect of loss of GSNOR1 on the internalization of PM-localized PIN2-GFP, the interference of the newly synthesized PIN2-GFP on the level of PM-localized PIN2-GFP must be excluded. To do so, we firstly treated the 6-day-old seedlings with cycloheximide (CHX; 50 μM), an inhibitor of *de novo* protein synthesis, and followed by washout with CHX plus BFA. As shown in Figure 3, the GFP intensity on the PM, the number of BFA bodies per cell and the relative GFP intensity of BFA bodies were all significantly reduced in *gsnor1-3* mutant seedlings relative to the wild-type cells after treatment with CHX for 30 min and followed by washout with CHX and BFA for additional 15 min or 60 min (Compare Figure 3A and Figure 3D; Figure 3B and Figure 3E; Figure 3C and Figure 3F; and see statistical data shown in Figures 3G–I). Consistent with the results obtained without CHX treatment (Figure 1I), the GFP intensity ratio of BFA bodies/PM was significantly reduced in the *gsnor1-3* mutant cells relative to the wild-type cells after CHX and BFA co-treatment (Figure 3J). Accordingly, the relative level of the PM-localized GFP signal was higher in the *gsnor1-3* than in Col-0 after CHX and BFA co-treatment (Figure 3K). These results again indicate that the PIN2-GFP internalization is impaired in the *gsnor1-3* even without *de novo* protein synthesis.

### The exogenously applied GSNO recapitulates the loss of GSNOR1 in inhibiting PIN2-GFP internalization

**Figure 4:**
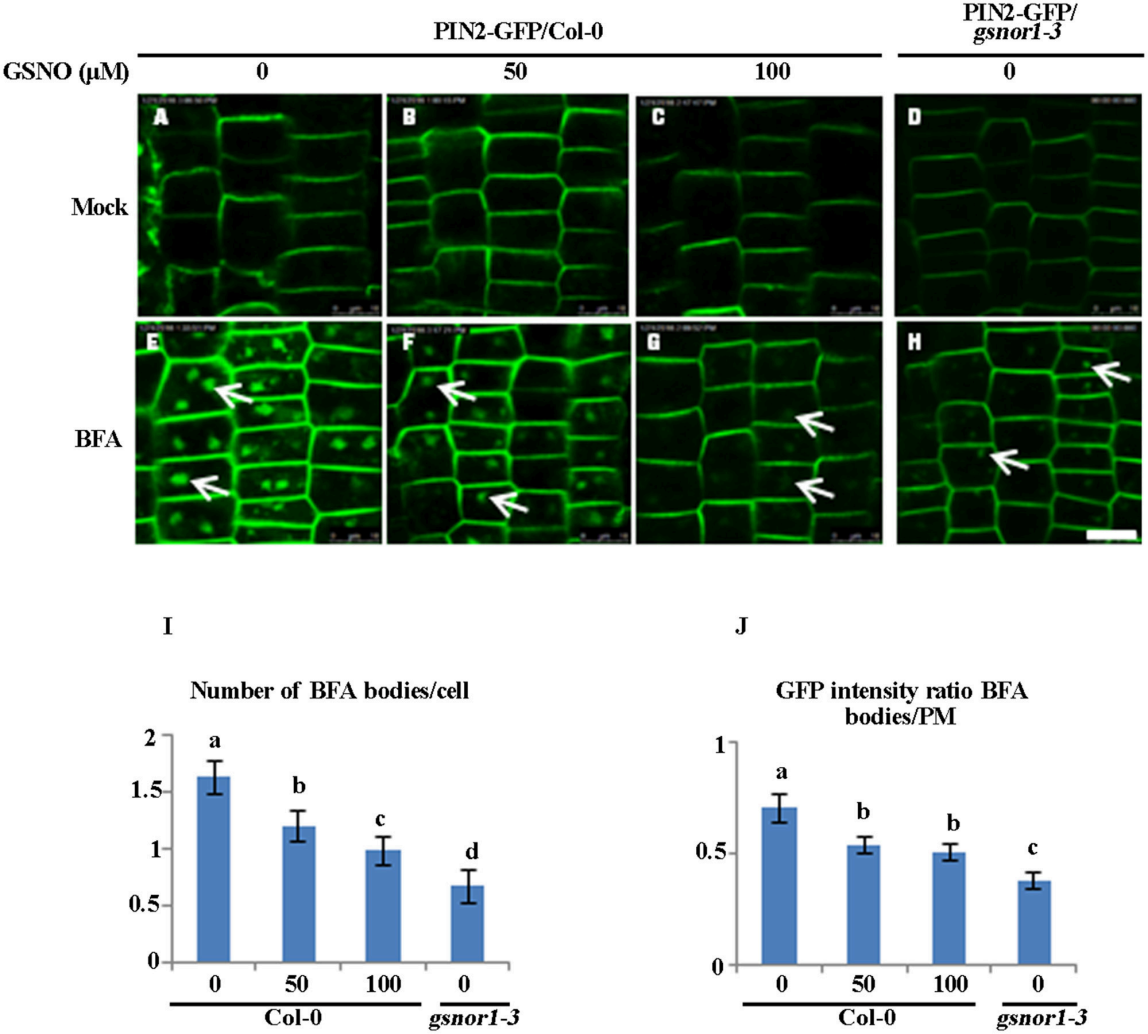
Exogenously applied GSNO reduces both the internalization and the PM-associated level of PIN2-GFP in the Col-0 seedlings in a concentration-dependent manner. The 2-day-old wild type Col-0 seedlings grown on ½ MS agar plates were transferred onto the agar plates containing different concentration of GSNO **(A–C)**, or different concentration of GSNO plus 50 μM BFA **(E–G)** for additional 4 day. The *gsnor1-3* mutant is used as a control **(D,H)**. The images were captured by confocal laser scanning microscopy (CLSM, Leica TCS SP5 AOBS). The numbers of BFA bodies (see white arrows) were counted **(I)** and the fluorescence intensities of the BFA bodies and at the PM were measured, respectively, using Image J (http://rsb.info.nih.gov/ij). The GFP intensity ratios of BFA bodies to PM were measured and the statistical data were presented in **(J)**. Bar = 50 μm. a, b, c, and d indicate significant differences between the treatments by Student's *t*-test.

We reasoned that if the impaired internalization of the PIN2-GFP or PIN2 observed in the *gsnor1-3* (Figures 1–3) is indeed resulted from over-accumulation of cellular SNO, the exogenously applied GSNO should have a similar inhibitory effect on PIN2 internalization. To test this hypothesis, we tested the effect of exogenous GSNO treatment on the PIN2-GFP internalization in the wild-type cells. Consistent with the results obtained using the *gsnor1-3* mutant (Figures 1, 2), exogenously applied GSNO not only reduced the level of the PM-localized PIN2-GFP (Figures 4A–C) but also inhibited the PIN2-GFP internalization in the presence of BFA in a concentration-dependent manner (Figures 4E–G, and statistical data in Figures 4I,J). These results suggest that the impaired polar auxin transport observed in the *gsnor1-3* mutant seedlings (Shi et al., 2015) could be resulted at least partially from the reduced internalization of PIN2 (Figures 4D,H–J).

### Exogenous GSNO inhibits root elongation in a concentration dependent manner

**Figure 5:**
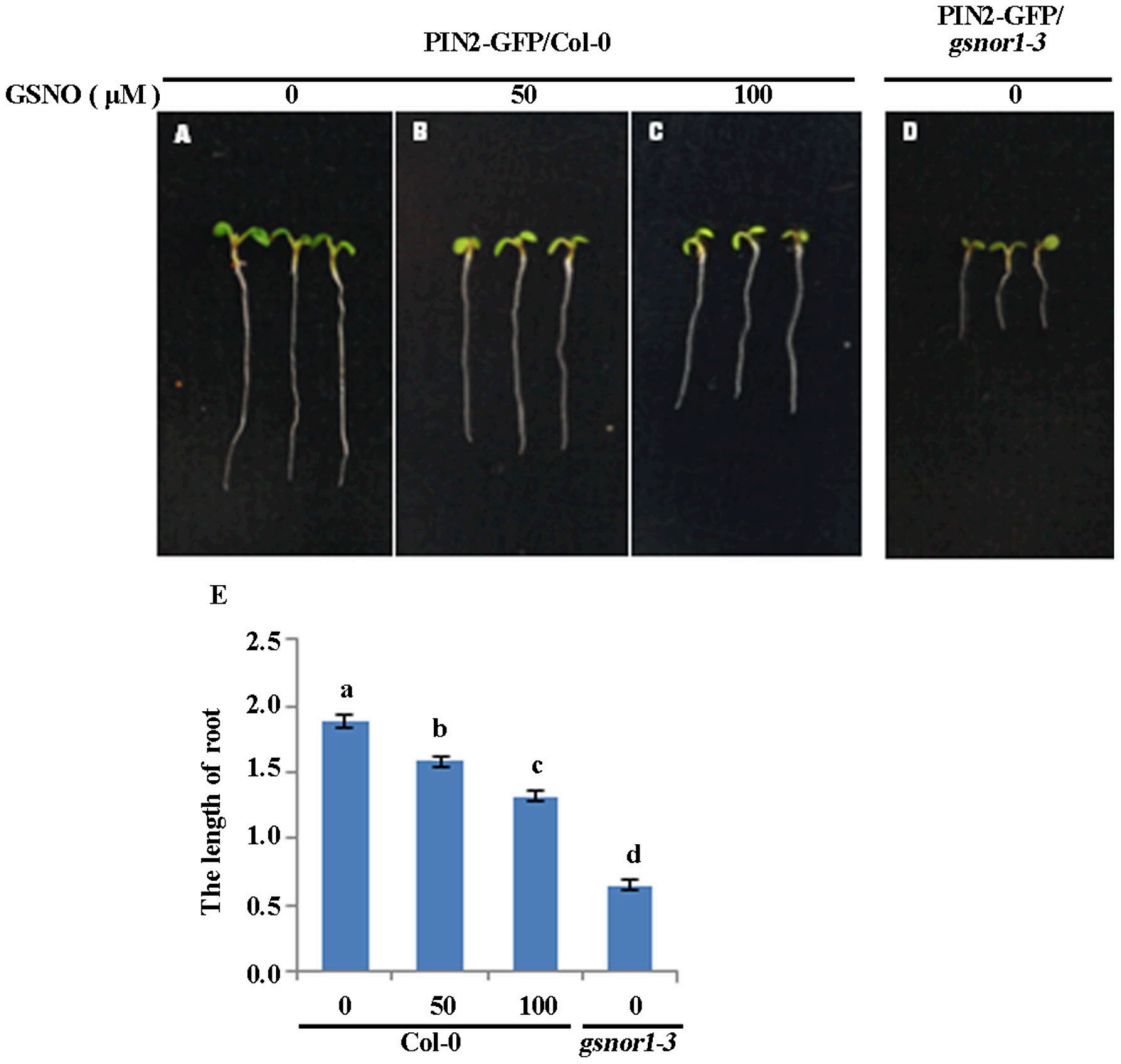
Exogenously applied GSNO inhibits the root elongation of the Col-0 seedlings in a concentration-dependent manner. The 2-day-old seedlings of wide type Col-0 grown on ½ MS agar plates were transferred onto the agar plates supplied with 0 **(A)**, 50 **(B)**, and 100 μM GSNO **(C)**. The *gsnor1-3* seedlings grown on the agar plates without containing GSNO used as a control **(D)**. The photos were taken 4 days after transfer. The root lengths were measured and listed in **(E)**. a, b, c, and d indicate significant differences between the treatments by Student's *t*-test.

Our previous studies have shown that loss of GSNOR1 impairs auxin signaling and transport and the *gsnor1-3* mutant plants display a wide range of auxin-related morphological defects including a short root phenotype (Shi et al., 2015). We postulated that if the short root phenotype observed in the *gsnor1-3* mutant seedlings is indeed a consequence of cellular SNO over-accumulation, the exogenously applied GSNO could mimic the effects of the loss-of-function mutant of GSNOR1. To test this postulation, we treated the Col-0 seedlings with different concentrations of GSNO and used the *gsnor1-3* mutant seedlings as a control. As expected, the exogenously applied GSNO inhibited the root elongation of the wild type seedlings in a concentration-dependent manner (Figures 5A–C), confirming that the excessive cellular SNO is at least partially, if not fully, responsible for the short root phenotype of the *gsnor1-3* mutant seedlings (Figures 5D,E, and Shi et al., 2015).

## Discussion

It is not an uncommon phenomenon that phytohormones play roles in regulating CME. It has been reported that strigolactones affect shoot branching by modulating the endocytosis of PIN1 (Shinohara et al., 2013), and salicylic acid (SA) represses endocytosis of different PM-associated proteins by blocking clathrin recruitment at the PM (Du et al., 2013). Auxin inhibits the CME of several PM-localized proteins, including several PIN proteins (Paciorek et al., 2005). As a result, auxin promotes its own efflux by inhibiting the internalization of PINs and increases various PIN levels at the PM (Paciorek et al., 2005). In this report, we provided evidence that NO, the other phytohormone, also play a role in the regulation of PIN2 endocytosis.

Our previous results have shown that even though the transcript level of *PIN2-GFP* was higher in the *gsnor1-3* mutant than in the wild type Col-0, the intensity of PIN2-GFP at the PM was significantly reduced in the *gsnor1-3* mutant relative to the wild-type plants (Shi et al., 2015). Likely, the reduced accumulation of PIN2 at the PM in the *gsnor1-3* could be partially resulted from compromised protein synthesis and/or stability and the reduced levels of the various PIN proteins in the *gsnor1-3* could be the primary cause of the compromised polar auxin transport (Shi et al., 2015). However, our present data uncover that, in addition to the reduced level of PIN2 at the PM, the internalizations of PIN2 was also compromised in the *gsnor1-3* mutant seedlings (Figures 1–3). Post-translational modifications play critical roles in regulating endocytosis. Both mono- and poly-ubiquitylation of single lysine is associated with cargo internalization and the intracellular sorting and targeting of PM proteins to the vacuole/lysosome rely on K63-linked ubiquitylation (Luschnig and Vert, 2014). PIN2 is modified by K63-linked poly-ubiquitin chains, which dependent on a class of ring-domain E3 ligase (RGLGs) (Yin et al., 2007; Leitner et al., 2012). Protein phosphorylation has been identified as a major determinant of PIN sorting and the sorting decision is dependent on the phosphorylation status of PINs and the activity of the serine/threonine protein kinase PINOID (PID) and its related proteins impact polar PIN distribution (Friml et al., 2004). As NO can also regulate protein functions by *S*-nitrosylation, it is highly possible that NO regulates internalization either directly by *S*-nitrosylating PINs or indirectly by *S*-nitrosylating the other key proteins in the endocytic pathways. This statement is supported by the facts that the activities of many PM-resident ion channels in animals, including Na^+^ and Ca^2+^ channels, are regulated by *S*-nitrosylation (Xu et al., 1998; Renganathan et al., 2002) and *S*-nitrosylation is invloved in modification of an outward-rectifying K^+^ channel in *Vicia faba* (Sokolovski and Blatt, 2004).

Theoretically, the relatively enhanced level of PIN2-GFP at PM could relatively enhance the auxin polar transport (Paciorek et al., 2005). However, we observed a much reduced polar auxin transport in the *gsnor1-3* mutant primarily because of the universally reduced levels of PINs (Shi et al., 2015). One possibility is that the positive contribution of the relatively enhanced PM-localized PIN2, as a result of impaired internalizations, to polar auxin transport is masked by the inhibition resulted from the overall reduced level of PIN2 (Figures 1, 2; Shi et al., 2015). The other possibility is that, even though the relative level of PM-localized PIN2 is enhanced in the *gsnor1-3* mutant relative to WT, the function of the PM-localized PIN2 is impaired under the excessive SNO condition, probably by *S*-nitrosylation. The functional endocytosis/exocytosis is required for replenishing the non-functional PM-localized PIN2 with newly synthesized functional PIN2 at PM. As a result, the reduced level of PIN2 and the compromised internalization of PIN2 (Figures 1–3) could contribute additively to the impaired polar auxin transport in the *gsnor1-3* mutant and thus its auxin-related morphological phenotypes.

Even though the auxin signaling is significantly impaired in the *gsnor1-3* (Shi et al., 2015), the auxin inhibition of either the transgenically expressed PIN2-GFP or the endogenous PIN2 internalization is not affected in the *gsnor1-3* (Figures 1C,F, 2C,F), suggesting that auxin signaling does not play a critical role in the inhibition of PIN2 internalization. In agreement with this, it has been shown that differential auxin regulation of CLC and CHC membrane association is ABP1-dependent but TIR1/AFB-independent (Wang et al., 2013) and the role of ABP1 in auxin signaling is questionable (Gao et al., 2015; Liu, 2015). Given that the clathrin are required for the auxin inhibition of the PIN2-GFP internalization (Wang et al., 2013) and the auxin inhibition of PIN2-GFP is not affected in *gsnor1-3* mutant (Figures 1C,F, 2C,F), it is suggested that the reduced internalization of PIN2-GFP or endogenous PIN2 (Figures 1, 2) may not be clathrin-dependent. In supporting this, it has been reported previously that the same PM protein could traffic through distinct endocytic routes (Beck et al., 2012). The activated FLS2 triggered by flg22 recognition at PM is targeted to the intracellular compartments for degradation via an ESCRT1-dependent, but BFA-insensitive route to prevent excessive and constitutive activation of defense signaling, whereas the non-activated or newly synthesized FLS2 is shuttled between PM and cytoplasm via a BFA-sensitive route (Robatzek et al., 2006; Beck et al., 2012; Spallek et al., 2013).

One may argue that the fluorescence intensity of some BFA bodies in *gsnor1-3* mutant (Figures 1E, 2E) could be below the detection threshold under our Confocal settings, and therefore could have an impact on the fluorescence intensity ratio of BFA bodies/PM in the mutant. If that is really the case, the average fluorescence intensity of the BFA bodies within a cell or in a given captured image (5–20 root cells/image) of *gsnor1-3* root should be lower, and thus the fluorescence intensity ratio of BFA bodies/PM in the mutant should be lower than shown in Figures 1J, 2J. As a result, the detection thresholds issue would not change our final conclusion that the internalization of either the transgenically expressed PIN2-GFP or the endogenous PIN2 was compromised in the *gsnor1-3* mutant.

In this report, we provided genetic and pharmaceutical evidence that the NO signaling plays an inhibitory role in PIN2 internalization. However, the molecular mechanism underlying the inhibition still remains unanswered. The questions like how NO signaling inhibits PIN2 internalization and what is the causal relationship between the NO-inhibited PIN2 internalization and polar auxin transport need to be addressed in the future. Nonetheless, our results reveal an additional layer of complex roles of NO in regulating plant growth and development through modulating internalization of auxin efflux transporter.

## Materials and methods

### Arabidopsis lines and growth conditions

Arabidopsis Col-0 and *gsnor1-3* (Feechan et al., 2005), the ProPIN2:PIN2-GFP transgenic line (Xu and Scheres, 2005) were used in this study. The ProPIN2:PIN2-GFP transgene was introgressed into the *gsnor1-3* background by crossing. The homozygous *gsnor1-3* line that expresses the *ProPIN2:PIN2-GFP* transgene was identified by the genomic PCR in combination of phenotype characterization among F3 population. These plants were grown under 16 h-light (22°C) /8h-dark (18°C). Seedlings were grown on 1/2 MS medium containing 1.0% sucrose (pH 5.7). Healthy six-day-old seedlings were used in this study.

### Chemical solutions

The stock solutions of CHX (50 mM, Sigma-Aldrich), BFA (50 mM; Invitrogen) and GSNO (100 mM, Sigma-Aldrich) were prepared in DMSO. 2,4-D (10 mM, Sigma-Aldrich) was firstly dissolved in 1 M KOH and then diluted with water as described (Wang et al., 2013).

### Chemical treatments

All chemical pretreatments except GSNO were for 30 min, and chemical treatment time is indicated in the text. For GSNO treatment, 2-day-old seedlings were transferred from GSNO-free 1/2 MS agar plates (containing 1.0% sucrose, pH 5.7) into agar plates containing different concentrations of GSNO as indicated in the text for additional 3-4 days. All seedlings were grown on MS basal salts with minimal organics (Sigma Aldrich) supplemented with 1% (w/v) Sucrose and 0.05% (w/v) MES-KOH, pH 5.6 (0.5 3 x MS), liquid medium, except otherwise specified. The final pH of the medium was 5.6–5.7.

### Polyclonal antibody and immunofluorescence microscopic analysis

Polyclonal antibody, anti-AtPIN2, was raised in rabbits as described using a synthesized peptide (Wang et al., 2013) coupled with keyhole limpet hemocyanin containing an additional N-terminal Cys (Huabio). Immunofluorescence microscopic analysis was performed as described (Wang et al., 2013). The primary PIN2 antibody was detected using Cy3-labeled anti-rabbit secondary antibodies (Sigma-Aldrich). Fluorescence images were captured using CLSM (Leica TCS SP5 AOBS). Excitation wavelengths for GFP and Cy3 were 488 nm (argon laser) and 561 nm (diode laser), respectively. The emission wavelengths were 496–532 nm for GFP and 550–570 nm for Cy3, respectively. For the purpose of direct comparisons of fluorescence intensities, laser, pinhole, and gain settings of the confocal microscope were set exactly identical when capturing the images from the seedlings of different genotypes or treatments. The intensities of fluorescence signals both at the PM and the cytoplasmic vesicles were quantified on the captured digital images using Image J (http://rsb.info.nih.gov/ij) and the relative fluorescence intensities were presented as percentages of mock controls as described (Sauer et al., 2006; Robert et al., 2010). For the measurement of the fluorescence levels at the PM, optimal sections of the root cells were used for measurements. Using Image-J, PM regions of a captured images (over 20 cells for Figure 1; 5–10 cells for Figures 2–4) were selected with the rectangle tool. The mean pixel intensity readings (total intensity readings/area) for the selected PM regions were recorded and the average values were calculated subsequently. The data shown in Figures 1, 3, 4 were averages from the different images captured at least from 30 individual roots. The data shown in Figure 2 were averages from the different images captured at least from 10 individual roots. The experiments were repeated three times with similar results.

For the measurement of the fluorescence levels at the BFA bodies, the regions of the visible BFA bodies in a single cell of the captured images (over 20 cells for Figure 1; 5–10 cells for Figures 2–4) were selected with the oval tool of Image J. The mean pixel intensity readings (total intensity readings/area) for the selected regions in a single cell were recorded and the mean value of the fluorescence intensity of the BFA bodies in a single cell was calculated by dividing the number of BFA bodies in the single cell (the sum of mean values/the number of BFA bodies). The average fluorescence intensity of the BFA bodies in a captured image were calculated subsequently by dividing the number of cells in the captured image (the sum of average fluorescence intensity of the BFA bodies in a single cell/the number of cells in a captured image). The data shown in Figures 1, 3, 4 were averages from different images captured from at least 30 individual roots. The data shown in Figure 2 were averages from the different images captured at least from 10 individual roots. The experiments were repeated three times with similar results.

For calculation of the fluorescence intensity ratio of BFA/PM, the average fluorescence intensity of the BFA bodies measured in a captured image was divided by the average fluorescence intensity at the PM of the same image. The data shown in Figures 1, 3, 4 were mean values of the images captured from at least 30 individual roots. The data shown in Figure 2 were the mean values from the images captured at least from 10 individual roots. The experiments were repeated three times with similar results.

The BFA-induced internalization of PM-localized proteins was presented as the average number of fluorescence-labeled BFA bodies per cell (Robert et al., 2010). For all the quantitative data, a Student's *t* test (paired with two-tailed distribution) was used in statistical analysis.

### Root elongation assay

The 2-day-old Col-0 seedlings grown on the 1/2 MS agar plates were transferred onto the 1/2 MS agar plates containing different concentration of GSNO for additional 3–4 days. The root length was measured under dissecting microscopy using Image J (http://rsb.info.nih.gov/ij).

## Author contributions

J-ZL and JP designed the experiments. MN, LZ, Y-FS, CW and YL performed the experiments. J-ZL and JW wrote the manuscript.

### Conflict of interest statement

The authors declare that the research was conducted in the absence of any commercial or financial relationships that could be construed as a potential conflict of interest. The handling Editor declared a shared affiliation, though no other collaboration, with several of the authors CW and JP.

## Abbreviations

2,4-D: 2,4-dichloropheenoxyacetic
CME: Clathrin-mediated endocytosis
CHX: cycloheximide
NO: Nitric oxide
GSNO: *S*-nitrosoglutathione
GSNOR1: *S*-nitrosoglutathione reductase 1
SNO: *S*-nitrosothiol
TGN: Trans-Golgi network.
